# Ascorbic acid is a dose-dependent inhibitor of adipocyte differentiation, probably by reducing cAMP pool

**DOI:** 10.3389/fcell.2014.00029

**Published:** 2014-08-07

**Authors:** Fryad Rahman, Fadi Al Frouh, Benoit Bordignon, Marc Fraterno, Jean-François Landrier, Franck Peiretti, Michel Fontes

**Affiliations:** ^1^Nutrition, Obesity and Risk of Thrombosis, INSERM U 1062, INRA 1260, Aix-Marseille UniversityMarseille, France; ^2^Service of Electron Microscope, Faculté de Médecine, Aix-Marseille UniversityMarseille, France

**Keywords:** ascorbic acid, adipocytes, cell differentiation, cyclic AMP

## Abstract

Ascorbic acid (AA) is the active component of vitamin C and antioxidant activity was long considered to be the primary molecular mechanism underlying the physiological actions of AA. We recently demonstrated that AA is a competitive inhibitor of adenylate cyclase, acting as a global regulator of intracellular cyclic adenosine monophosphate (cAMP) levels. Our study, therefore, aimed to determine new targets of AA that would account for its potential effect on signal transduction, particularly during cell differentiation. We demonstrated that AA is an inhibitor of pre-adipocyte cell line differentiation, with a dose-dependent effect. Additionally, we describe the impact of AA on the expression of genes involved in adipogenesis and/or the adipocyte phenotype. Moreover, our data suggest that treatment with AA partially reverses lipid accumulation in mature adipocytes. These properties likely reflect the function of AA as a global regulator of the cAMP pool, since an analog of AA without any antioxidant properties elicited the same effect. Additionally, we demonstrated that AA inhibits adipogenesis in OP9 mesenchymal cell line and drives the differentiation of this line toward osteogenesis. Finally, our data suggest that the intracellular transporter SVCT2 is involved in these processes and may act as a receptor for AA.

## Introduction

We have previously demonstrated that treatment of a mouse model of Charcot-Marie-Tooth 1A disease, an inherited peripheral neuropathy, with high doses of ascorbic acid (AA) at least partly reversed the phenotype of transgenic mice (Passage et al., 2004). In subsequent work, we demonstrated that the intracellular pool of cyclic adenosine monophosphate (cAMP) decreased when cells were incubated with increasing concentrations of AA (Kaya et al., 2007). This inhibition is specific to AA, and is not elicited by other antioxidants (Kaya et al., 2008b). Using classic enzyme inhibition experiments, we demonstrated that AA is a competitive inhibitor of adenylate cyclase (Kaya et al., 2008a). Through this intracellular effect, AA suppresses the expression of genes that are under the control of the cAMP signaling pathway. Additionally, we demonstrated that AA suppresses cell proliferation *in vitro*, as well as *in vivo* (Belin et al., 2009). Taken together, these studies suggest that AA could be an important factor in the regulation of cell differentiation through its novel function as a regulator of the intracellular cAMP levels. This novel perspective led us to investigate the cellular targets of AA that underlie the observed effects, especially those affected during cell differentiation.

Adipose tissue is mainly composed of adipocytes that accumulate lipid droplets in the cytoplasm. Moreover, adipocytes secrete a number of physiologically important molecules, including cytokines. Pre-adipocytes, fibroblast-like cells, differentiate from mesenchymal cells that are in turn derived from the mesoderm, an embryonic cellular layer that is also a progenitor of striated muscle and bones. Differentiation of pre-adipocytes into mature adipocytes has been studied using various mesenchymal fibroblast-like cell lines, with the majority of studies utilizing the 3T3-L1 cell line.

Increase in body mass index (BMI) is seen as a consequence of multiple factors, including obesity (genetic and/or nutritional), age, and environment, among others. Elevated BMI is associated with an increased risk of a number of disorders, mainly cardio-vascular, that shorten the life expectancy. In addition to its major role in lipid storage, adipose tissue functions as an endocrine organ, influencing metabolism (Spiegelman and Flier, 2001).

Multiple clinical studies report that plasma AA concentration is inversely related to BMI and fat distribution (Canoy et al., 2005; Johnston et al., 2007; Aasheim et al., 2008). Additionally, AA has been reported to interfere with lipid accumulation in animals fed a high-fat diet (Campión et al., 2006), but no mechanism has been proposed to explain these findings. Two broad molecular mechanisms may account for this effect; one involves the action of AA on biochemical processes linked to metabolism, while another putative mechanism involves the effect of AA on tissue dynamics. Our hypothesis is that AA may be involved in the second process and affect adipocyte differentiation. The study described in this manuscript, therefore, has the following goals:

1. To evaluate the impact of AA on pre-adipocyte differentiation. Addition of AA to the cell incubation medium has been long recognized to impact cell differentiation processes (myelin formation, osteogenesis, and others), but the mechanism is largely unknown at this time and active doses are yet to be determined.
2. To provide information on the signaling pathways involved in the effect of AA on differentiation.
3. To elucidate how the AA signal is transduced to the cell. No AA receptor has been identified so far. Could a molecule such as the transporter SVCT2 play this role?

The gene expression cascade activated during adipocyte differentiation has been largely described. A number of transcriptional factors were identified as being involved in terminal differentiation of adipocytes, including peroxisome proliferator-activated receptor γ (PPAR-γ) (Tontonoz et al., 1994; Spiegelman and Flier, 2001; Puigserver and Spiegelman, 2003), CCAAT-enhancer-binding proteins (C/EBP) α and β (Umek et al., 1991; Yeh et al., 1995), and sterol regulatory element-binding protein 1 (SREBP-1c) (Kim and Spiegelman, 1996; Le Lay et al., 2002). PPARγ and C/EBP expression are stimulated early in the pre-adipocyte differentiation process. Genes such as adiponectin are expressed later, as part of the terminal differentiation processes (Musri et al., 2010). However, the role of non-protein factors that may be driving cell differentiation is poorly understood.

In this article, we demonstrate that AA inhibits adipocyte differentiation in a dose-dependent manner. Moreover, treatment with AA partially reverses the differentiation of preadipocytes to mature adipocytes. Additionally, we demonstrate that AA inhibits adipogenesis in OP9 cell line. Finally, we demonstrate that expression of sodium-dependent vitamin C transporter 2 (SVCT2), a transporter protein and a putative AA receptor, is probably involved in these differentiation processes. Our findings suggest that these intracellular effects are not linked to the antioxidant properties of AA, since they also occur following treatment with an analog without any antioxidant capacity that is capable of modulating cAMP levels.

## Materials and methods

### Cells culture

Mouse 3T3-L1 cells were obtained from ATCC. Pre-adipocytes cells were cultured in DMEM containing 25 mmol/l glucose and supplemented with glutamax and without pyruvate, 10% (v/v) FBS, 1% penicillin/streptomycin (100 U/ml and 100 μg/ml), and 1% biotin/pantothenate. The cells were inoculated into either 6 multidishes at a density of 2 × 10^4^ cells/well for gene expression assays or 96 wells micro plate at a density of 5 × 10^3^ to evaluate lipid accumulation. Cells are maintained at 37°C under a humidified 5% CO_2_ atmosphere. The numbers of cells were evaluated with a Neubauer hemocytometer, using 0.05% trypsin-EDTA dissociated cells, and cell viability was monitored by means of the trypan blue-dye exclusion test.

OP9 cells were cultured in MEM-α containing 20% FBS, 1% L-glutamine (2 mM), 1% penicillin and Streptomycin at 37°C in humid field containing 5% of CO_2_. Cells are let to spontaneously differentiate during 2 weeks in DMEM + dexamethasone at 10^−7^ mol/L with or without AA at 100 μM.

### Adipocytes differentiation

Cells were cultured for 9 days in DMEM containing 25 mmol/l glucose, 10% (v/v) FBS, antibiotics and supplemented with dexamethasone (1 μmol/L), and insulin (1 μg/ml). IBMX was added or not to the medium. AA was added or not to the medium (dissolved in 1× PBS and then filtered). Medium was changed every 2 days.

### Qualitative analysis of lipid accumulation

Cells were rinsed by PBS and fixed with 10% (v/v) buffered formalin in Dulbecco's PBS for 10 min. Then formalin discarded and 2 ml of fresh formalin was added and incubate for 24 h. Following fixation, cells were washed by ddH_2_O twice. The cells were let to dry at RT. Following drying, Oil Red-O solution (60% in distilled water) was added and cells incubated for 10 min. Cells were rinsed twice with ddH_2_O. Pictures were acquired under the microscope.

### AdipoRed™ assays

Cells were seeded at a density of 5 × 10^3^ cells/well (96-wells plate), and then cultured in DMEM containing 10% FBS and 1% antibiotics and treated with dexamethasone (1 μmol/l), insulin (1 μg/ml), and various concentration of AA for 9 days. Thereafter, cells were rinsed with PBS. Then AdipoRed™ (Lonza Walkersville, USA) a dye that binds lipids, and, in different wells, SYBR^®^ Green I (Life Technology), that binds DNA (as an evaluation of cells number), were added. Plates were then read on a microplate reader (VICTOR™X4 from PerkinElmer^®^) and the fluorescence of AdipoRed™ and SYBR^®^ Green I were measured with excitation 485 nm/emission 572 nm and excitation 494 nm/emission 521 nm respectively. Adipored fluorescence was normalized to DNA content.

### Von Kossa staining

Cell cultures were fixed in cold methanol for 15–20 min. After rinsing, the fixed cells were incubated with (5%) Silver Nitrate Solution (abcam product # ab150687) and crosslinked under UV light for 20–30 min, then rinsed twice with distilled water. The dishes were incubated with (5%) Sodium Thiosulfate Solution for 2–3 min, rinsed twice with distilled water; then finally incubated with Nuclear Fast Red Solution for 5 min, and rinsed several times with distilled water to remove excess stain.

### Evaluation of cAMP concentration

Intracellular cAMP level was measured using cAMP-Glo™ Assay (Promega Corp.). Briefly, 100 μl of 3T3L1 cells solution (5 × 10^4^ cells/ml) was deposited in a sterile 96-wells microplate (culture treated) and incubated at 37°C in 5% CO_2_/95% air. After cell adhesion (24 h), culture medium was replaced by 20 μl of our compounds solutions, prepared in “induction buffer.” Each solution dilution was tested in triplicate, and wells containing 3T3L1 cells in 20 μl of induction buffer alone were used as controls like basal level of intracellular cAMP. Then, assay was performed according to the manufacturer's instructions. At the end, plates were read using a microplate reader (Victor™ X4, PerkinElmer^®^). According to kit instructions, “Induction Buffer” was composed with PBS containing 500 μM of 3-isobutyl-1-methylxanthine (IBMX) and 100 μM of 4-(3-butoxy-4-methoxybenzyl)imidazolidin-2-one (Ro20-1724). The IBMX and Ro20-1724 are inhibitors of phosphodiesterases and used to prevent cAMP hydrolysis during the assay. In summary, this assay evaluates cAMP production, as a consequence of adenylate cyclase activity.

### Quantitative evaluation of lipids and mineralization in cells cultures

Pictures were acquired for stained cells in culture using a Zeiss microscope. Presence of mineralized nodules and deposited calcium were seen as dark brown to black spots using Von Kossa staining methods, while lipid accumulation droplets are colored in red by Oil Red O or appear as white droplet spots. The evaluation of lipid droplets and mineralization were quantified by means of ImageJ software version 1.47. Area presenting brown coloration, red coloration or white droplets is evaluated in fields of 7 mm^2^. Ten fields have been evaluated.

### Gene expression assays

Total RNA was extracted from cells using TRIZOL reagent (Invitrogen™) according to the manufacturer's protocol. Purified total RNA from adipocytes was reverse transcribed using Superscript II reverse transcriptase (Invitrogen™) to produce cDNA. Thereafter, quantitative real time-polymerase chain reaction (PCR) was performed using Light Cycler 480 Real-Time PCR System (Roche), using UPL probes. Specific primers were used and described in Table 1. Expression levels of target genes were normalized using β-actin (ACTB) as internal standard. The results were treated using the comparative C_T_ method, where the amount of the target, normalized to the endogenous reference and relative to a calibrator, is given by 2^−ΔΔCT^.

**Table 1:** **Specific primers used in qPCR experiments**.

**RNA**	**Primer**	**Sequences (5′ → 3′)**
C/EBPα	Forward	AGCAACGAGTACCGGGTACG
	Reverse	GTTTGGCTTTATCTCGGCTC
C/EBPβ	Forward	AAGATGCGCAACCTGGAG
	Reverse	CAGGGTGCTGAGCTCTCG
Srebf1	Forward	GGTTTTGAACGACATCGAAGA
	Reverse	CGGGAAGTCACTGTCTTGGT
PPARγ	Forward	GAAAGACAACGGACAAATCACC
	Reverse	GGGGGTGATATGTTTGAACTTG
SVCT2	Forward	TGTGCCAGGCTCTCCTGT
	Reverse	GACCCTCCACGAAAATACCC
Adiponectin	Forward	TCCTGGAGAGAAGGGAGAGAAAG
	Reverse	CAGCTCCTGTCATTCCAACAT
TiMP3	Forward	GCCTCAAGCTAGAAGTCAACAAA
	Reverse	TGTACATCTTGCCTTCATACACG
β-actin	Forward	CTAAGGCCAACCGTGAAAAG
	Reverse	ACCAGAGGCATACAGGGACA

### Statistical analysis

We used Prism v5 software to perform statistical analysis of data. Data corresponding to an experiment were analyzed using ranking tests (Wilcoxon and Kruskal–Wallis). Results presenting a *p*-value < 0.05 were further analyzed using the Mann–Whitney two-tailed statistical significance test, with a confidence interval of 95%. We considered a *p*-value lower than 0.05 as significant. IC_50_ has been evaluated using the dedicated section of Prism software.

## Results

### AA inhibits differentiation of mesenchymal 3T3 L1 cells into adipocytes

**Figure 1:**
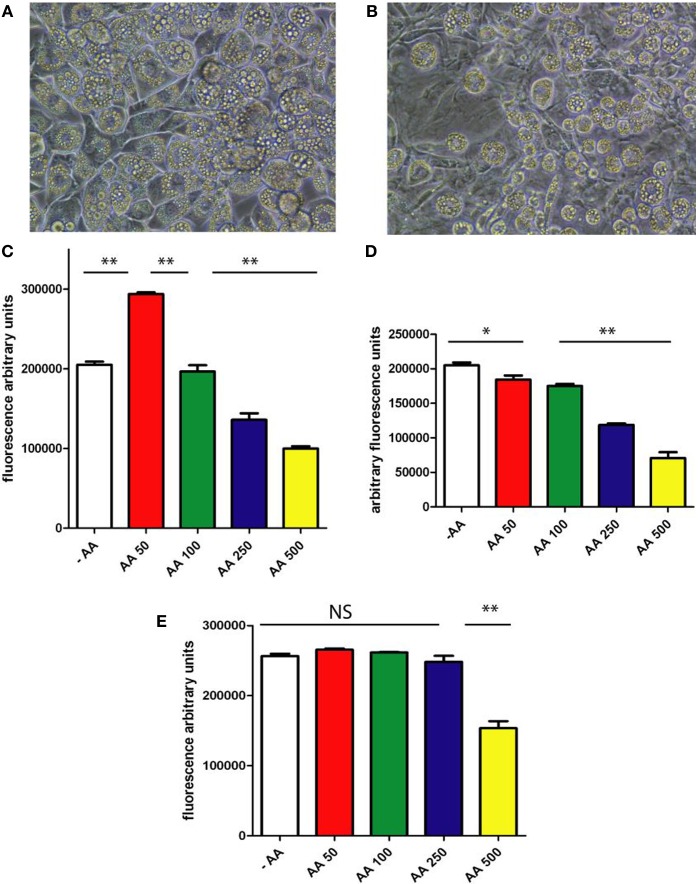
**3T3L1 differentiation**. Cells were cultured, with IBMX, during 9 days without AA **(A)** or with 0.5 mM of AA **(B)**. After 9 days cells are stained with Oil Red O. **(C)** In an independent experiment 3T3L1 cells were treated with IBMX during 3 days and cultured without AA and with increasing doses of AA during a total of 9 days. At the end, AdipoRed is added and fluorescence recorded (for details see Materials and Methods). Results are expressed in fluorescence arbitrary units. **(D)** Cells were treated during 3 days with IBMX and with increasing doses of AA. Cells were then allowed to differentiate without AA. At the end of the experience (9 days from the starting point of the experiment) lipids accumulation is recorded using AdipoRed assay (see Materials and Methods). **(E)** Cells were incubated with IBMX during 3 days without AA. Cells were then incubated without IBMX and with increasing doses of AA. Lipids accumulation was recording, using AdipoRed assay (see Materials and Methods) after 9 days from the starting point of the experiment. Schematic representation of our protocol is presented in Supplementary Figure 1. ^*^p < 0.05, ^**^p < 0.01.

In a first experiment, we cultured 3T3 L1 cells for 9 days in a medium that promotes adipocyte differentiation with IBMX, with and without AA. We observed that cells treated with 500 μmol/L AA present with less fully differentiated adipocytes, as depicted in Figures 1A,B. The experiment was repeated using increasing concentrations of AA to establish the dose-dependence of this effect. At low concentration of AA (50 μmol/L), we observed an increase in lipid accumulation (Figure 1C). However, when concentration of AA increased, we observed inhibition of lipid accumulation. This result could be explained by the competition between the effects of IBMX and AA. Since IBMX increases cAMP levels (by inhibiting phosphodiesterases), while AA inhibits cAMP production (Kaya et al., 2007, 2008a), it becomes obvious that an increase in AA concentrations counteracts the effect of IBMX. In order to determine if there is a critical period for AA action, cells were treated for 3 days with IBMX and AA at a range of concentrations (Figure 1D) and left to differentiate until day 9. We observed that 3 days of treatment were sufficient to inhibit lipids accumulation. In a separate experiment, cells were treated for 3 days with IBMX without any AA, followed by incubation without IBMX, but with increasing doses of AA. We observed (Figure 1E) that lipid accumulation in cells that underwent this treatment was not inhibited by AA, except at a concentration of 500 μmol/L. A schematic representation of these experiments is presented in Supplementary Figure 1. Our findings suggest the existence of a critical period for AA action, extending from day 0–3.

**Figure 2:**
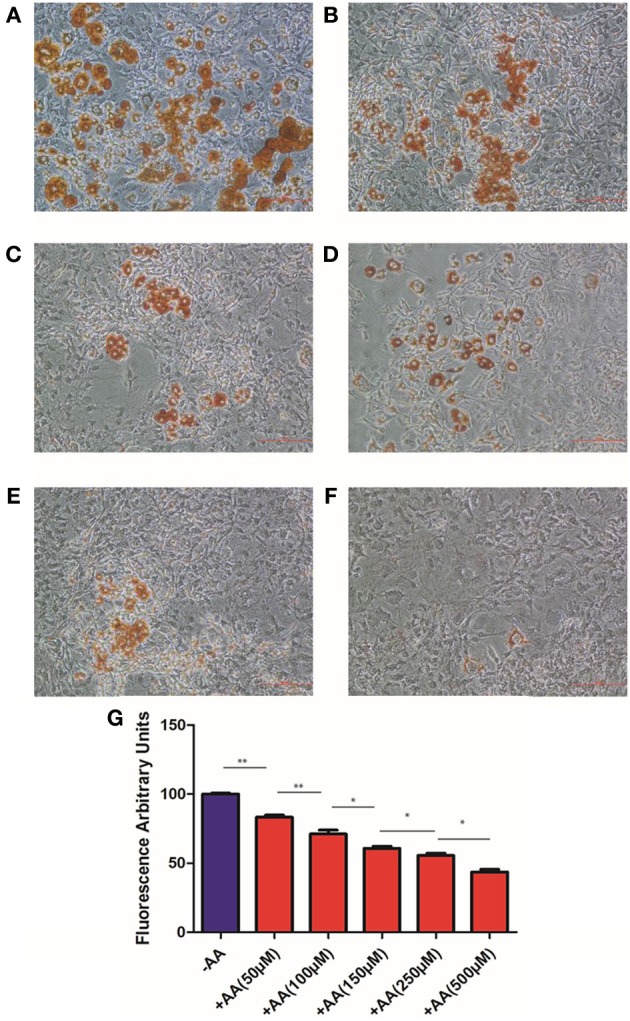
**3T3L1 differentiation**. Cells were cultured, without IBMX, during 9 days without AA **(A)** or with 50 μM **(B)**, 100 μM **(C)**, 150 μM **(D)**, 250 μM **(E)**, and 500 μM **(F)**. After 9 days cells were stained with Oil Red O. **(G)** In an independent experiment 3T3-L1 cells were cultured with or without AA. After 9 days, AdipoRed was added and fluorescence recorded (for details see Materials and Methods). Results are expressed in fluorescence arbitrary units. ^*^p < 0.05, ^**^p < 0.01.

In order to avoid competition between the IBMX-induced increase in cAMP levels, and AA-mediated decrease, we cultured 3T3 L1 pre-adipocytes in a medium without IBMX for 9 days with or without AA. In these conditions, the percentage of cells that differentiated into mature adipocytes was found to be lower, but we did observe that cells could differentiate into adipocytes in cultures without AA (Figure 2A). The next question was to determine whether inhibition of lipids accumulation by AA is dose-dependent. As presented in Figures 2A–F, we observed that lipid accumulation is inhibited in a dose-dependent manner when cells are incubated with increasing doses of AA. In a further experiment, cells were cultured in 96-well microplates in a medium without IBMX and with increasing doses of AA. After 9 days, lipid accumulation was evaluated using an assay developed in our laboratory (see Materials and Methods), with the results presented in Figure 2G. These data demonstrated that inhibition of 3T3 L1 cells differentiation by AA is dose-dependent, with IC_50_ about 150 μmol/L.

### Expression of specific markers of adipocyte differentiation is inhibited by AA

**Figure 3:**
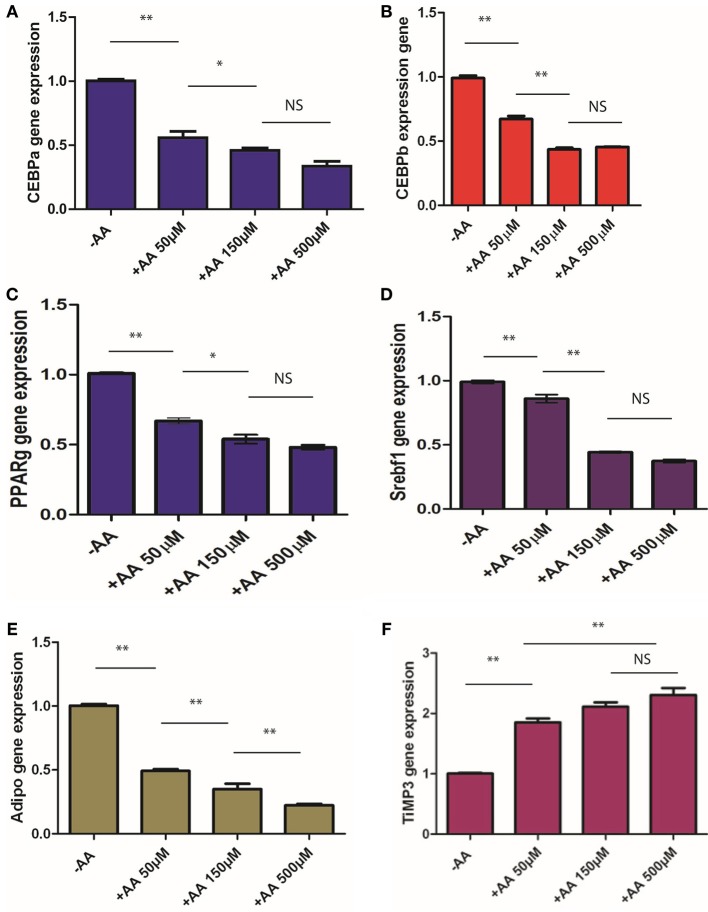
**Markers expression**. 3T3 L1 were cultured without IBMX and without or with different concentrations of AA. RNAs were extracted after 9 days and expression of different genes, CEBPα **(A)**, CEBPβ **(B)**, PPARγ **(C)**, SREBf1 **(D)**, Adiponectin **(E)**, and TIMP3 **(F)** have been evaluated using qPCR (see Materials and Methods). Basic levels, expression without incubation with AA is set to 1 and relative levels evaluated. ^*^p < 0.05, ^**^p < 0.01.

3T3-L1 cells were cultured for 9 days in a medium without IBMX with a range of concentrations of AA. RNA was extracted and expression of specific markers of adipocyte differentiation evaluated by qPCR. Results are presented in Figure 3. We observed that expression of genes associated with adipocyte differentiation (CEBP-α and -β, PPAR-γ, and SREB) was lower in the cells treated with AA compared to that in the cells incubated without AA, and this inhibition was dose-dependent. Expression of adiponectin, a marker of late stages of differentiation, was also inhibited by AA. Additionally, expression of TIMP3, which is normally reduced during adipocyte differentiation (Bernot et al., 2010), was elevated in cells treated with AA (Figure 3F). Expression of these differentiation markers is in agreement with the proposed inhibitory effect of AA on adipocyte differentiation.

### AA suppresses the accumulation of lipids in mature adipocytes

**Figure 4:**
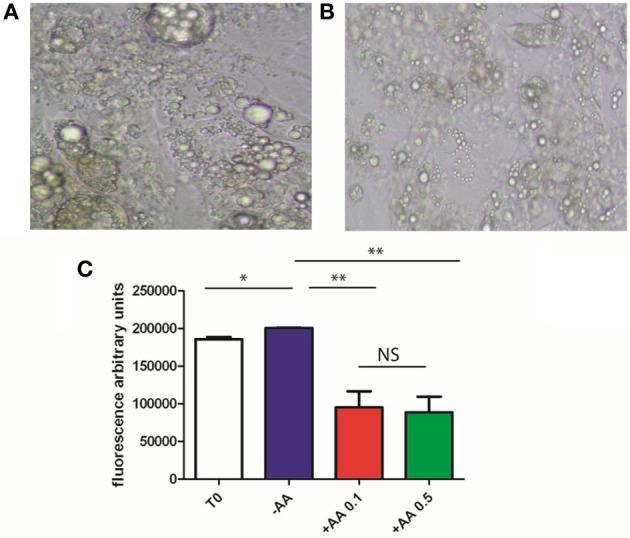
**3T3L1 and dedifferentiation**. Cells were cultured during 7 days with IBMX, allowing full differentiation. Medium is discarded and replaced with a medium without IBMX either not supplemented **(A)** or supplemented with 0.1 mM AA or 0.5 mM **(B)**. After 9 days, AdipoRed was added and fluorescence recorded (for details see Materials and Methods), **(C)**. Results are expressed in fluorescence arbitrary units. ^*^p < 0.05, ^**^p < 0.01.

We demonstrated above that AA inhibits differentiation of mesenchymal cells into mature adipocytes. To gain information regarding the action of AA on mature adipocytes, we cultured 3T3-L1 cells in a medium allowing their differentiation into mature adipocytes, in the presence of IBMX. When maturation was observed following 7 days of incubation, the cells were incubated in a medium without IBMX and the culture continued for 7 days with or without AA (0.1 of 0.5 mmol/L). At the end of the final incubation, lipid accumulation was recorded. As presented in Figures 4A–C, we observed that lipid accumulation continued in the absence of AA, but was decreased when AA is added to the medium.

These results suggest that AA is able to act on mature adipocytes, reversing lipid accumulation. However, it will be interesting to confirm these results using stromal vascular fraction (SVF) of adipose tissue and testing lipid accumulation and gene expression.

### A derivative of AA devoid of antioxidant properties also inhibits adipogenesis

**Figure 5:**
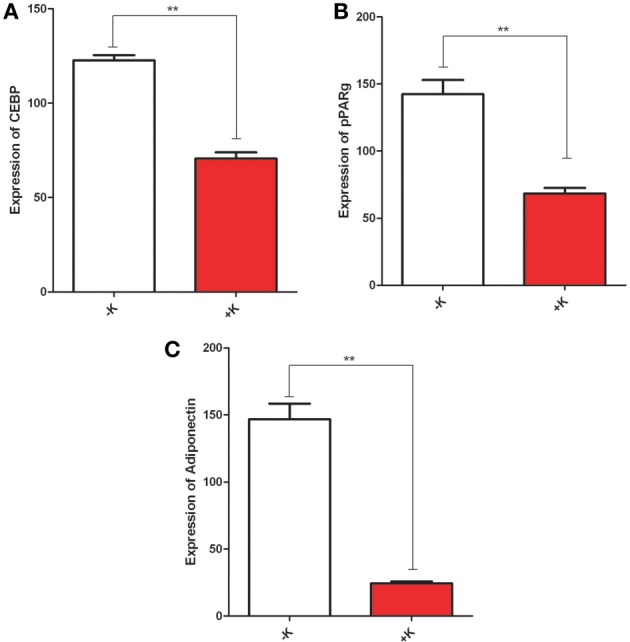
**Treatment of preadipocytes with K873, an analog of AA**. 3T3 L1 were incubated without or with AA during 9 days in an adipogenic medium. RNAs have been extracted and expression of CEBPβ **(A)**, PPARγ **(B)**, and adiponectin **(C)** was evaluated. ^**^p < 0.01.

AA has been described primarily as an antioxidant, but our recent work (Kaya et al., 2007, 2008a,b) identified a new function: a global regulator of cAMP levels through competitive inhibition of adenylate cyclase. To investigate whether the observed effect of AA on cell differentiation was due to its direct action on the cAMP pool or an outcome of its antioxidant properties, we used K873, an analog of AA recently synthesized in our laboratory. This molecule did not present any antioxidant properties but, like AA, it was shown to reduce the cAMP levels, although at lower concentrations (Bordignon et al., 2013). Treatment of 3T3-L1 with K873 during adipocyte differentiation inhibited the expression of adipocyte markers (C/EBPβ, PPAR-γ, and adiponectin), as observed in Figure 5. This result indicates that inhibition of adipocyte differentiation by treatment of pre-adipocytes with K873 is likely to result from its effect on cAMP levels.

### Cyclic AMP is involved in the effects of AA and K873

**Figure 6:**
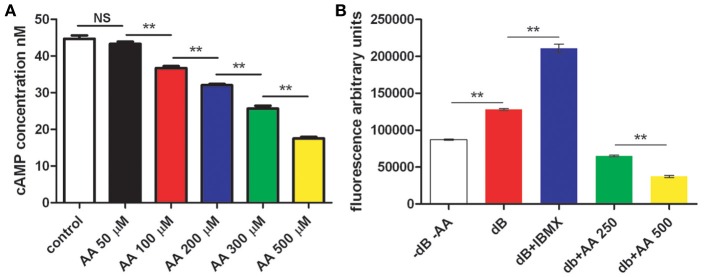
**cAMP, AA, and adipocyte differentiation. (A)** 3T3L1 have been incubated without and with AA at increasing concentration. At the end cAMP concentration was evaluated as described (see Materials and Methods). **(B)** 3T3L1 cells were treated either with dibutyrylcAMP (dBcAMP), dBcAMP+IBMX, or dBcAMP plus AA at two doses. Lipids accumulation has been evaluated using adipored technique (see Materials and Methods). Fluorescence has been recorded. ^**^p < 0.01.

While we have previously described a reduction of cAMP levels following treatment with AA in different cell types, this effect has never been evaluated in mesenchymal pre-adipocytes. We tested 3T3L1 incubated without AA or with increasing concentrations of AA, and evaluated the intracellular cAMP concentration. Results are presented in Figure 6A. We observed that AA decreased intracellular cAMP concentration in a dose-dependent manner. Moreover, we incubated 3T3L1 with dibutyril-cAMP (db-cAMP) alone or in the presence of IBMX without adding AA. We observed that addition of these compounds increased lipid accumulation, as was previously shown. Additionally, we treated 3T3L1 cells with db-cAMP and added AA to the medium. Adding AA inhibited lipid accumulation stimulated by db-cAMP (Figure 6B).

Taken together, our data confirm that increasing intracellular cAMP concentration promotes adipogenesis, while lowering intracellular cAMP levels through treatment with AA inhibits adipogenesis.

### Treatment with AA inhibits differentiation of OP9 cells into adipocytes, but drives it toward osteogenesis

**Figure 7:**
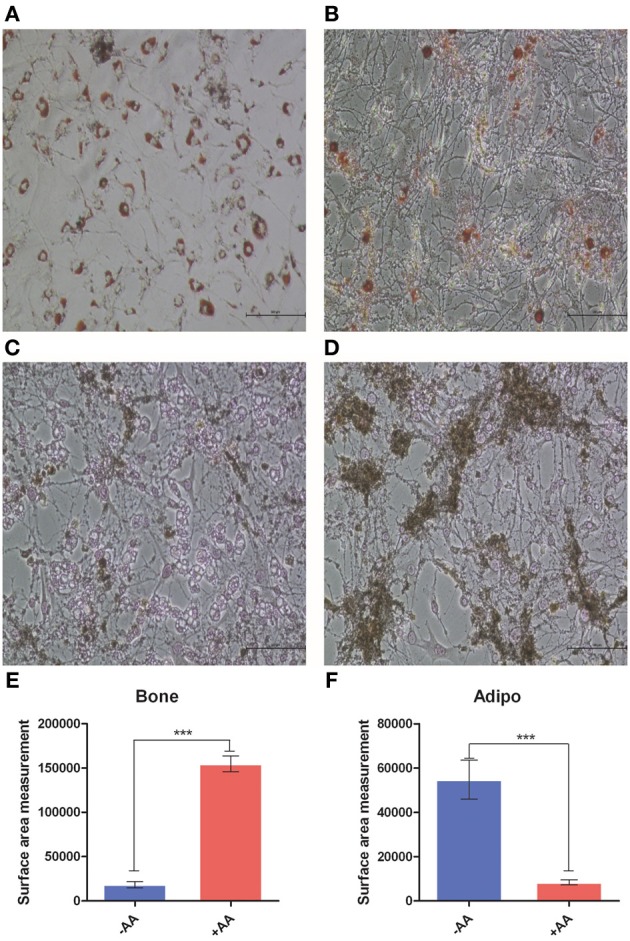
**OP9 cell differentiation**. OP9 cells were cultured in a medium (see Materials and Methods) without AA **(A,C)** or with AA **(B,D)**. After 21 days, cells have been stained either with Oil Red O **(A,B)** or with Von Kossa Staining **(C,D)**. Pictures have been captured using a Zeiss light microscope. Surface of fields presenting Von Kossa staining **(E)** or Oil Red O staining **(F)**, was evaluated using ImageJ software package. ^***^p < 0.001.

In order to rule out the possibility that the observed properties of AA are specifically linked to the 3T3 L1 cell line, we analyzed the effect of AA on differentiation of OP9 cells. This cell line has adipogenic capacity, but could also differentiate into osteogenic cells, depending on the culture conditions (Gao et al., 2010). In addition to evaluating the impact of AA on adipogenic differentiation, this analysis provided us with data regarding the impact of AA on osteogenic differentiation, another mesodermic lineage. We treated OP9 cells with a medium promoting adipocyte differentiation, with or without AA. After 21 days of culture, cells were stained either with Oil Red O to label lipids or with Von Kossa staining to highlight osteogenic differentiation. As shown in Figure 7, we observed that OP9 cells without AA treatment differentiate into adipocytes. Conversely, cells treated with AA poorly differentiated into adipocytes but exhibited clear signs of osteogenic differentiation. These data suggests that AA drives adult mesodermic mesenchymal cells to differentiate following the osteogenic pathway and inhibits adipogenic differentiation.

### SVCT2, the intracellular transporter of AA, is involved in its effects

**Figure 8:**
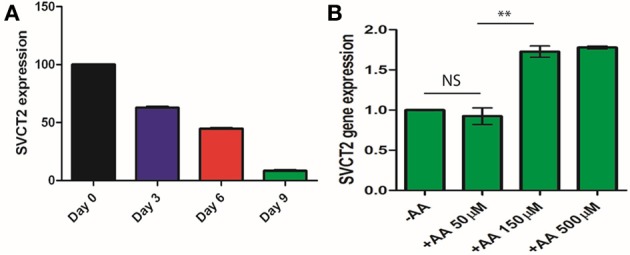
**Expression of SVCT2 during 3T3L1 differentiation. (A)** Cells were cultured in a medium with IBMX, allowing full differentiation, without AA. RNAs are extracted after 3, 6, and 9 days of culture. Expression of SVCT2 is evaluated using qPCR and specific primers (see Table 1) **(B)**. Adipocytes have been cultured in a medium without IBMX, as described in Figure 3, and with increasing concentrations of AA. Expression of SVCT2 was evaluated using qPCR. ^**^p < 0.01.

Little is known about a putative receptor of AA. However, the presence of the transport/receptor of AA, SVCT2, was shown to be required for AA signaling in various conditions (Sotiriou et al., 2002). SVCT2 is a transmembrane protein present in a number of cell types that exhibits high specificity for AA and could act as its receptor. Therefore, we evaluated the expression of the gene coding for this protein during adipocyte differentiation. We observed that the expression of this gene decreased during differentiation (Figure 8A), with almost no expression of this gene detected in mature adipocytes. Additionally, we observed that treatment with AA increased SVCT2 expression in a dose-dependent manner (Figure 8B). Several articles (Reidling et al., 2008; Wu et al., 2008; Bordignon et al., 2013; Hong et al., 2013) report that a decrease in gene expression is correlated with a decrease in concentration of the corresponding protein SVCT2. In addition, these articles report that variations in expression of the gene coding for SVCT2 have an impact on various biological processes.

In conclusion, mature adipocytes, under normal conditions, could probably not receive signals from AA present outside cells. Pre-adipocytes treated with AA, however, continue to receive the AA signal, as evidenced by the removal of repression of SVCT2 expression.

## Discussion

Several publications suggest that AA may be involved in cell differentiation, including a study that investigated Schwann cell differentiation and myelination. This work demonstrated that AA should be added to Schwann cells/axons co-culture to induce myelin formation. Moreover, we demonstrated that high concentrations of AA promote remyelination in a mouse model of CMT type 1A disease (Passage et al., 2004). A second line of research has been recently initiated and investigates the differentiation of embryonic stem cells (ES). Using high-throughput screening, Takahashi et al. identified AA as the only molecule present in the searched chemical library that was able to promote differentiation of murine ES into cardiomyocytes (Takahashi et al., 2003).

Finally, Sotiriou et al. described a phenotype of a genetic mouse model in which the SVCT2 gene has been invalidated. When homozygotic cells are placed in an environment with normal outside AA concentration, the molecule is unable to enter the cells. Embryos died in a perinatal period, with lung and blood vessel anomalies noted (Sotiriou et al., 2002). In addition, Gess et al. demonstrated that heterozygotes in this model exhibit a defect in myelination and present a CMT-like phenotype (Gess et al., 2011). These observations provide the rationale for investigating the role of AA as a signaling molecule modulating development and differentiation of mammals.

AA was demonstrated to be essential for the stimulation of myelin formation in osteogenesis. In our current study, we demonstrate that AA inhibits the differentiation of mesenchymal cells into mature adipocytes. Two independent cell lines have been used in this investigation (3T3 L1 and OP9), with the same results. Our observations appear to contradict previously published reports, but the differences in outcomes could be explained by the approaches used. Firstly, several papers did not use AA itself, but rather its phosphate salts (Ono et al., 1990; Choi et al., 2008). In one publication that did use AA (Weiser et al., 2009), the experiments described were performed at a low concentration (50 μmol/L) in bone marrow-derived cells. In our current manuscript, we show slight stimulation of differentiation to adipocytes of pre-adipocytes treated with IBMX (a molecule that increases intracellular cAMP concentration) and a low concentration of AA (an inhibitor of adenylate cyclase). However, increasing concentrations of AA led to an inhibition of differentiation to adipocytes, likely as a result of a competition between IBMX and AA, with increasing concentrations of AA suppressing the effect of IBMX. In order to avoid the competition between IBMX and AA, the effect of AA was also assessed on the “spontaneous” differentiation to adipocytes, without IBMX stimulation. In these conditions, although differentiation is less efficient, we show that AA inhibits the differentiation to adipocytes with IC_50_ about 150 μmol/L.

In a further step, we evaluated the expression of specific markers of differentiation. We observed that AA inhibits the expression of genes (C/EBP, SREB, PPAR-γ, and adiponectin) involved in different stages of adipocyte differentiation (Kim et al., 2010). Regulation of these genes shares a common feature, with their expression being under direct or indirect control by cAMP and cAMP-dependent pathways (Kim et al., 2010). This finding confirms previously published data demonstrating that adipocyte differentiation is driven by cAMP-dependent pathways (Jia et al., 2012; Kadota et al., 2012). Additionally, a recent publication (Zhang et al., 2012) demonstrated that high intracellular cAMP concentration drives ES cell differentiation toward adipocytes. Conversely, low cAMP concentration inhibits adipocyte differentiation and promotes differentiation of ES cells to osteoblasts (Kao et al., 2012). This modulation is of particular interest, considering our demonstration that AA, as a competitive inhibitor of adenylate cyclase, is a regulator of cAMP levels (Kaya et al., 2008a) and lowers intracellular concentration of cAMP at increasing concentrations. Our findings are further validated by the inhibition of adipogenesis by K873, an analog of AA without any antioxidant properties that modulates intracellular cAMP levels (Bordignon et al., 2013). Additionally, we demonstrated in current study that stimulation of adipogenesis by db-cAMP and IBMX is inhibited by treatment with AA at concentrations we have shown to be effective in reducing cAMP levels. It is thus likely that AA directly affects adipogenesis by modulating intracellular cAMP levels.

An interesting further question is what is the effect of AA on other lineages? We provide data showing that in addition to inhibiting adipogenic differentiation, AA promotes osteogenic differentiation. Kao et al. (2012) have demonstrated that cAMP signaling directs the differentiation of mesenchymal cells into osteoblasts, rather than adipocytes. We observe the same phenomenon with AA, a modulator of cAMP levels.

Finally, we observed that AA counteracts the repression of expression of SVCT2 (the transmembrane transporter of AA) that is known to take place during adipocyte differentiation. This phenomenon could allow cells to receive the AA signal leading to the repression of adipocytes differentiation. SVCT2 may therefore function as an AA receptor, with AA treatment increasing the expression of the SVCT2 gene. This suggests that repression of SVCT2 expression during the differentiation of pre-adipocytes into mature adipocytes is partially reversed by AA treatment, allowing cells to receive the AA signal and leading to a repression of adipocyte differentiation.

In addition, we have shown that treatment of mature adipocytes with AA prevents lipid accumulation. At this point, it is not possible to determine whether this observation reveals dedifferentiation of mature adipocytes, lipolysis, or a novel unknown mechanism. However, the observation is interesting, as it suggests that lipid accumulation in mature adipocytes could be modulated by AA treatment.

Finally, present data could explain why animals fed a high-fat diet accumulate fewer lipids when treated with AA (Campión et al., 2006). In addition, these observations could help us understand why low AA plasma level, in humans, are correlated with obesity and fat distribution (Canoy et al., 2005; Johnston et al., 2007; Aasheim et al., 2008). The last question to be addressed in the evaluation of AA effect would be whether the concentrations of AA necessary for the inhibition of adipocyte differentiation (IC_50_ 100 μmol/L) could be achieved by oral administration. During a clinical trial evaluating the effects of treatments with CMT1A and AA, we measured blood AA concentrations in participants divided into 3 treatment arms (60 in each arm) and treated during one year. Patients treated with placebo had an average concentration of 50 μmol/L, patients treated with 1 g/day had an average concentration of 80 μmol/L, while patients treated with 3 g/day had an average concentration of 106 μmol/L, reaching up to 160 μmol/L in some subjects (Micallef et al., 2009). A consensus of other studies is that oral supplementation with increasing doses of AA does not result in a corresponding increase in blood levels, since the excess of AA is excreted. This conclusion is based on numerous results of short-term postprandial experiments. However, our clinical data suggest that long-term supplementation with increasing doses increases blood levels of AA. While it is not clear why the findings with long-term treatment contradict the results of short-term experiments, our data suggest that AA concentrations in blood could be increased through oral supplementation. In addition, our clinical study has also shown that there are no adverse effects associated with treatment of patients with 3 g/day dose of AA, as compared to the placebo treatment, demonstrating that administration of this dose is safe for human use.

AA, as well as its derivatives, could therefore modulate cell signaling, with properties that could be of interest for applications in human health.

### Conflict of interest statement

The authors declare that the research was conducted in the absence of any commercial or financial relationships that could be construed as a potential conflict of interest.
