# Supplementary material for: Ascorbic acid is a dose-dependent inhibitor of adipocyte differentiation, probably by reducing cAMP pool
Source: Front Cell Dev Biol. 2014 Aug 7;2:29. doi: 10.3389/fcell.2014.00029 (PMC4207035; doi:10.3389/fcell.2014.00029)
Supplement: Supplementary file 1 [file Presentation1.PDF]

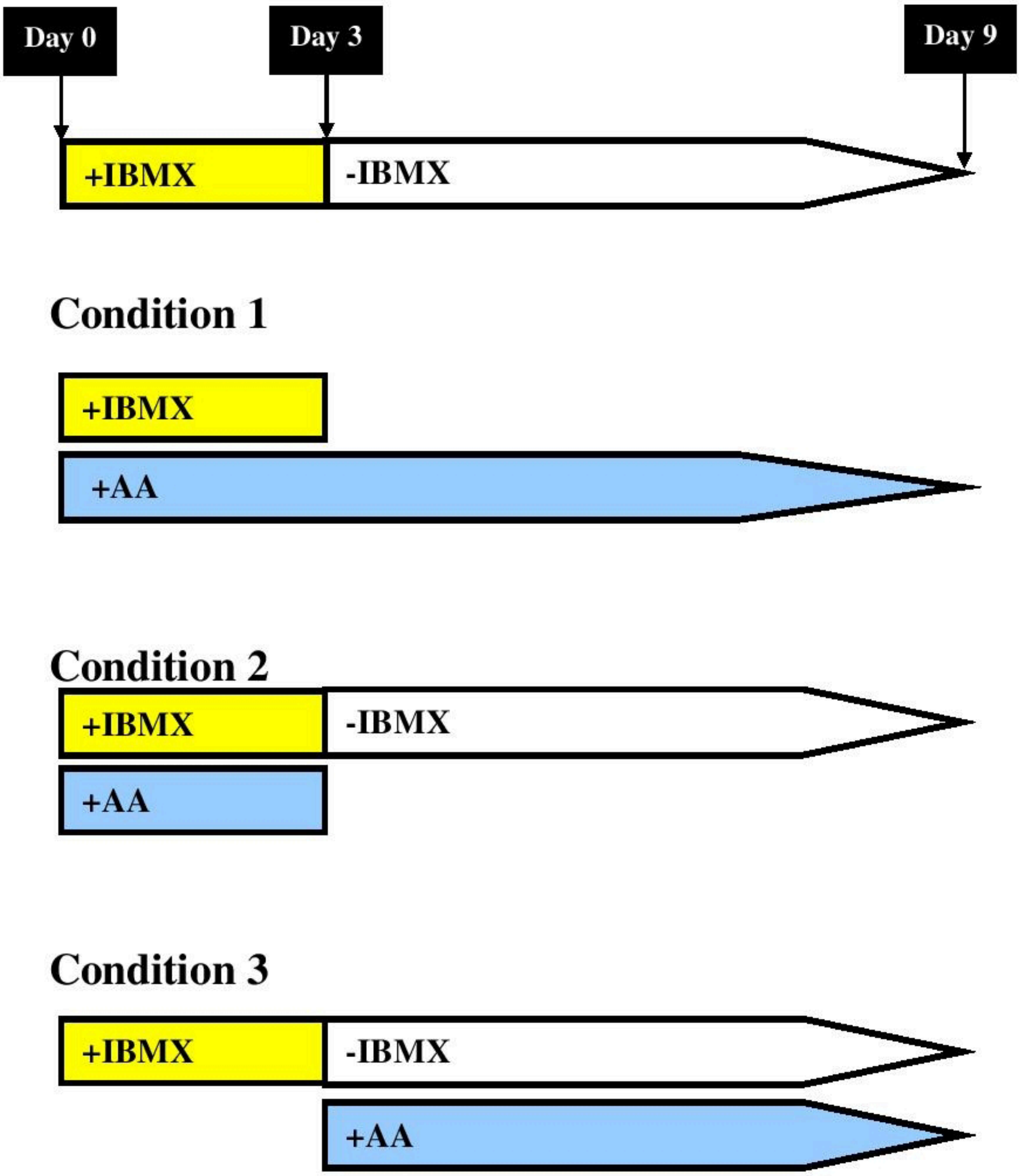

Supplementary figure 1. This figure summarizes the protocol used in experiments presented in figure 1. Periods of treatment with IBMX are indicated in yellow. Periods of treatment with AA are in blue and period of treatment without IBMX are in white. Condition 1 corresponds to figure 1C, condition 2 to figure 1D and condition 3 to figure 1E.
